# Supplementary material for: A novel multi-modal retrieval framework for tracking vehicles using natural language descriptions
Source: PLoS One. 2025 Aug 11;20(8):e0327468. doi: 10.1371/journal.pone.0327468 (PMC12338829; doi:10.1371/journal.pone.0327468)
Supplement: Attachment — Submitted filename: Response to Reviewers.pdf [file pone.0327468.s001.pdf]

Authors' Response of PONE-D-24-43459.R1

Changghao Zhang, Zhandong Liu*, Ke Li, Yong Li, Xiangwei Qi, Nan Ding,

February 14, 2025

To Associate Editor and Reviewers

We sincerely thank all reviewers for their valuable comments and suggestions, which have significantly contributed to enhancing the quality of our paper. We have diligently reviewed and addressed each comment, particularly focusing on the feedback from experts in machine learning and intelligent transportation systems. Major revisions are highlighted in blue, reflecting these essential changes.

To Associate Editor

Thank you for considering our manuscript for publication in PLOS ONE. We appreciate your constructive feedback and guidance.

Q1: Thank you for stating the following financial disclosure:

1) National Natural Science Foundation of China (Grant No. 62162061 and Grant No. 62262066).
2) Xinjiang Normal University Doctoral Initiation Fund Project (Grant No. XJNUBS2115).
3) Xinjiang Normal University Youth Top Talents Project (Grant No. XJNUQB2022-21).
4) Xinjiang Key Research and Development Program (2022B01007-1)

Please state what role the funders took in the study.

A1: Thanks for pointing out this issue. This is a very good question. Regarding the roles of the funders in the paper, we would like to clarify as follows:

1) National Natural Science Foundation of China (Grant No. 62162061 and Grant No. 62262066).
   These grants supported core research activities, including technology development, equipment procurement, dataset construction, annotation, and algorithm validation.
2) Xinjiang Normal University Doctoral Initiation Fund Project (Grant No. XJNUBS2115). This project provided start-up funds for preliminary research activities such as literature review and small-scale experiments, laying a solid foundation for further studies.

3) Xinjiang Normal University Youth Top Talents Project (Grant No. XJNUQB2022-21). This project offered additional financial support for exploring innovative aspects within the multi-modal retrieval framework, enabling us to focus on core issues and achieve breakthrough progress.

4) Xinjiang Key Research and Development Program (2022B01007-1)
   This program provided targeted research and development support, especially for developing vehicle tracking technology using natural language descriptions in specific Xinjiang scenarios.

Q2: Please remove any funding-related text from the manuscript and let us know how you would like to update your Funding Statement.

A2: Thanks for your insightful suggestion. In the revision, we have removed any funding-related text from the manuscript.

To Reviewer 1

We are grateful for your careful review and valuable support.

Q1: Section: Introduction, First Paragraph: The term "continuous recording" is vague—does it refer to constant monitoring or uninterrupted data collection?

A1: Thanks for pointing out this issue. This is a very good question. By "continuous recording," we mean uninterrupted data collection by the cameras deployed in the intelligent transportation system. This implies that the cameras are constantly capturing video footage without significant interruptions, enabling long-term monitoring of vehicle movements. We have clarified this in the revised manuscript.
"In intelligent transportation systems, cameras are deployed for uninterrupted data collection, resulting in a continuous stream of video footage. This continuous recording leads to a large-scale candidate pool for vehicle retrieval, which is a vital component of AI-driven smart city initiatives."

Q2: Section: Introduction, Second Paragraph: Typographical error. Missing the author(s) before "*et al.*"

A2: Thanks for your insightful suggestion. We have corrected the reference to include the author names before "*et al.*" in the revised manuscript.

Q3: Section: Frame Analysis, First Paragraph: "track ID and a corresponding bounding box." How are these generated? Are they automatically extracted via a tracking algorithm (*e.g.* DeepSORT)?

A3: Yes, the track ID and the corresponding bounding box are automatically extracted via a tracking algorithm, such as DeepSORT. This algorithm is capable of assigning unique track IDs to objects (in this case, vehicles or other entities within the video clips) and generating bounding boxes around them in each frame of the video clips. For the "Section: Frame Analysis, First Paragraph," we have made revisions in the revised manuscript as follows:
"Video frame information is a key component of the text-to-video module and has a significant impact on the retrieval process. Each camera video is divided into multiple video clips. To facilitate the tracking and identification of objects within these clips, we employ a tracking algorithm, such as DeepSORT, which automatically assigns a unique track ID to each object

and generates a corresponding bounding box around it in each frame of the video clips. This process ensures accurate and efficient object tracking throughout the video retrieval process."

Q4: Section: Frame Analysis, Figure 3: Figure 3 suffers from incomplete text, making it challenging to interpret. Please ensure all text and visual elements are legible and contained within the image boundaries.

A4: Thank you for bringing this to our attention. We have updated Figure 3 to ensure all text and visual elements are clear and fully contained within the image boundaries.
"In the video recognition model, a clean background of the local road is generated by computing the median value of each pixel across all frames in the video. Utilizing this background, a brief video clip can be created, which incorporates a mask for the region of interest (ROI) and highlights the vehicle corresponding to the specified track ID. To further enhance image quality and boost the model's robustness, a random number is chosen to dictate the frame interval for each iteration. An illustration of a processed video frame is provided in the left corner of Figure 1."

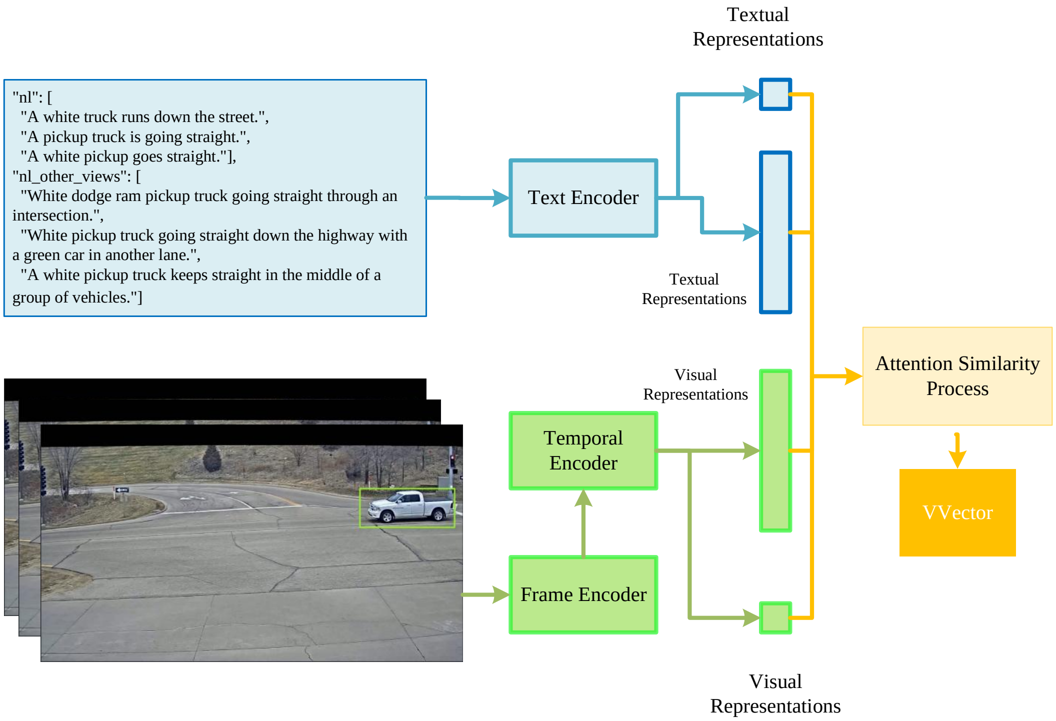

Figure 1: The core architecture of our video recognition module involves different processing steps for visual and textual data. Video frames are fed into a frame encoder(Vision Transformer) and a temporal encoder to generate visual representations. Meanwhile, textual information is processed through a text encoder(Text Transformer [1]) to generate corresponding textual representations. These visual and textual representations are then combined via an attention similarity mechanism to predict a score for model evaluation and updating.

Q5: Section: Pre-processing, Fourth Paragraph: While clustering synonyms (*e.g.*, "brown" and "beige") reduces diversity, it may oversimplify semantic nuances. How does this clustering handle edge cases or ambiguous words?

A5: Thanks for pointing out this issue. This is a very good question. We acknowledge that clustering synonyms can indeed oversimplify semantic nuances. To handle edge cases or ambiguous words, we use a semantic similarity threshold to determine when words should be clustered. If a word falls below this threshold, it is considered too ambiguous to be clustered and is left unchanged. We have explained this process in the revised manuscript.
"To further address the diversity issue in text descriptions, we apply the same semantic similarity threshold principle. Words are clustered based on their semantic similarity, but only if they exceed the threshold. For example, while 'brown' and 'beige' may be considered synonyms, we only cluster them if their similarity score meets or exceeds the threshold. If a word's similarity score falls below the threshold and it is deemed too ambiguous, it remains unchanged. Additionally, to standardize the description of vehicle motion trajectories, we have categorized them into four types: straight, left turn, right turn, and stop, as detailed in Table 1."

Table 1: Vehicle natural language description lables from CityFlow-NL dataset

**Class**	**Labels**
Color	blue, brown, gray, orange, black, purple, silver, green, white, yellow, red
Type	sedan, SUV, pickup, van, bus, truck
Motion	straight, stop, left, right

Q6: Baseline Vehicle Video-Text Retrieval Module, Equation 2: The formulas for " $V(v_i, t_j)$" and "$S(v_i, t_{jk})$" are unclear in terms of practical implementation. For example, how are $S_{vs}, S_{vw}, S_{fs}, S_{fw}$ calculated?

A6: Thank you for your insightful comments and questions regarding the clarity of the formulas in our paper. We appreciate the opportunity to provide further details on the practical implementation of the equations, particularly focusing on how $S_{vs}, S_{vw}, S_{fs}, S_{fw}$ are calculated.

In our original submission, we briefly mentioned that these variables are generated by the baseline vehicle video-text retrieval module. To enhance clarity and ensure reproducibility, we have expanded on this section to include a more detailed description of the calculation process.
"In addition, $S\left(v_i, t_{jk}\right)$ can be expressed as:

$$S\left(v_i, t_{jk}\right) = w_{jk} \times \left(S_{vs} + S_{vw} + S_{fs} + S_{fw}\right)/4, \tag{1}$$

where w_{jk} indicates the text ID_j assigned to the *k-th* natural language description. If this description belongs to the corresponding natural language description, the associated weight will be higher; otherwise, if it represents a different natural language perspective, the weight will be lower. The variables S_{vs}, S_{vw}, S_{fs}, and S_{fw} represent the video-sentence score, video-word score, frame-sentence score and frame-word score, respectively. These scores are generated by the baseline vehicle video-text retrieval module through the following processes:

- Video-Sentence Score S_{vs}: This score is calculated by measuring the semantic similarity between the entire video content and the given sentence. Techniques such as cosine similarity on feature vectors extracted from a pre-trained video-language model can be employed.
- Video-Word Score S_{vw}: This score assesses the relevance of individual words in the sentence to the video content. It can be computed by averaging the similarity scores between each word in the sentence and the video features, possibly using word embeddings and video frame embeddings.
- Frame-Sentence Score, S_{fs}: Similar to S_{vs}, this score measures the semantic similarity between each frame of the video and the entire sentence. This can be achieved by computing the similarity for each frame-sentence pair and then averaging these similarities.
- Frame-Word Score S_{fw}: This score evaluates the relevance of individual words to specific frames in the video. It is calculated by averaging the similarity scores between each word and each frame, utilizing word embeddings and frame-level features.

By integrating these scores, the baseline module provides a comprehensive assessment of the video-text relevance, which is then weighted by w_{jk} to reflect the importance of the natural language description in the context of the retrieval task."

A7: Section: Vehicle Motion Module, First Paragraph: The text mentions "a set of threshold parameters" but does not specify how these thresholds are chosen or tuned. Please provide more detail about this process.

A7: Thank you for your meticulous review and for bringing this important point to our attention. We appreciate your suggestion to provide more detail regarding the selection and tuning of the threshold parameters in the Vehicle Motion Module.
We have revised the paragraph to include a detailed explanation of the methodology used to determine these thresholds. This includes a description of the empirical testing process and any optimization techniques that were employed. Below is the revised text:

Vehicle Motion Module

"This module is dedicated to analyzing vehicle maneuvering trajectories with the aim of developing advanced directional control systems. By utilizing the given trajectory, which comprises a series of bounding boxes, we derive the starting vector of the vehicle by applying linear regression to the initial segment of the trajectory's center points. Similarly, the ending vector is obtained by fitting the center points of the final segment using another linear regression function. The angle between these two vectors is then calculated to establish a reference point. To determine the final direction, a set of threshold parameters is assumed.
These threshold parameters are chosen through an empirical testing process combined with optimization techniques. Initial threshold values are selected based on domain knowledge and preliminary experiments. Subsequently, these thresholds are fine-tuned using cross-validation methods on a labeled dataset containing various vehicle maneuvers. Specifically, we use k-fold cross-validation to evaluate different threshold settings and choose the one that minimizes classification error while maintaining robustness across different types of vehicle movements. For example, if the angle falls between -10 and 5 degrees, the vehicle is classified as moving

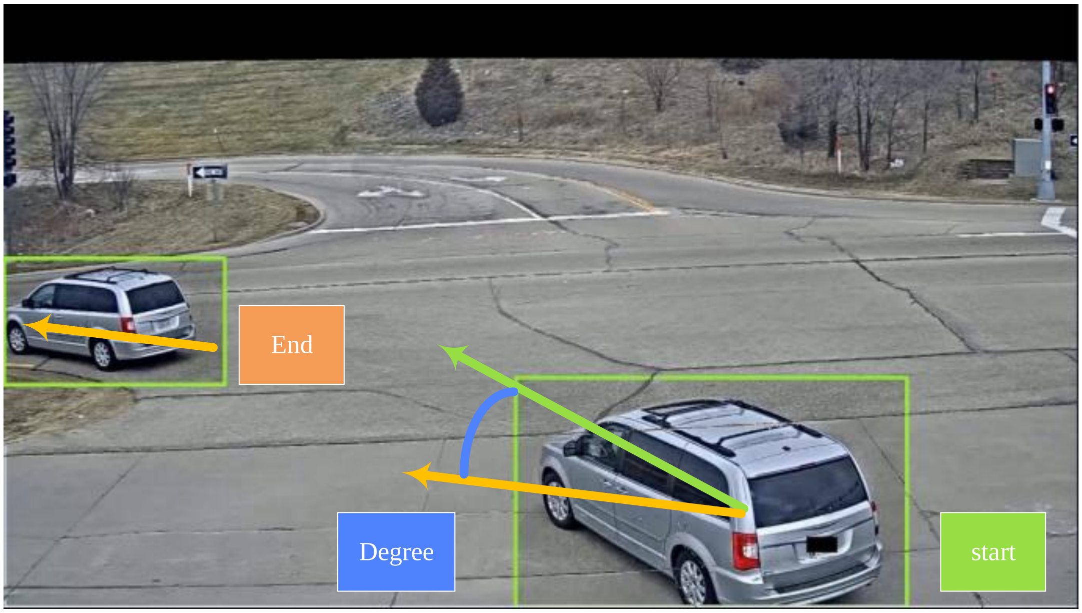

Figure 2: Example of a vehicle motion module. In the figure, the starting vector is represented by a green arrow, the ending vector is represented by an orange arrow, and the angle between the two vectors is represented by a blue arc.

straight. The process is illustrated in an intuitive manner in Figure 2, which clearly outlines the approach we adopt in the vehicle motion module."

Q8: Section: Post-processing, Equation 3: The formula for V_w divides by 2, but it's unclear if this is normalization or an arbitrary scaling factor. If it's normalization, why was this particular factor chosen instead of another method (*e.g.*, accounting for vector norms)?

$$V^w = \left(w_1^w \times \sum_{i=1}^{3} V_i^v + w_2^w \times \max_{i=1}^{n} V_i^v \right) \times \frac{1}{2}, \tag{2}$$

A8: Thank you for raising this important question regarding the scaling factor in Equation 3 for V_w. The division by 2 in the equation is indeed not intended as a normalization step in the traditional sense, where normalization typically involves scaling data to a specific range (e.g., unit norm) based on the data's intrinsic properties. Instead, the factor of is used as a simple averaging mechanism to combine the contributions from the two weighted components in a balanced manner.

The rationale behind choosing $\frac{1}{2}$ as the scaling factor is to provide an equal weight (after applying w_1^w and w_2^w) to the summed average of the first three text-video pairs and the maximum vector among all pairs. This approach aims to strike a balance between capturing the general trend from the primary NL descriptions (through averaging) and highlighting the most salient information from all descriptions (through taking the maximum).

We recognize that other methods, such as normalizing based on vector norms, could also be considered. However, in our specific context, we found that a simple averaging approach with a fixed scaling factor provided a straightforward and intuitive way to combine these two types of information without introducing additional complexity or assumptions about the relative importance of different vector norms.

To clarify this point in the paper, we have revised the relevant section as follows:
"In this subsection, we focus on the post-processing and result fusion of the MVR model. The model generates vector outputs from two different modules, which are then weighted and fused to create a comprehensive vector. The weighted vector is denoted as V^w and is calculated using the following formula:

$$V^w = \left(w_1^w \times \sum_{i=1}^{3} V_i^v + w_2^w \times \max_{i=1}^{n} V_i^v \right) \times \frac{1}{2}, \tag{3}$$

where V_i^v represents the *i-th* text-video pair vector, while w_1^w and w_2^w are the weights associated with the natural language (NL) description and all descriptions, respectively. These weights w_1^w and w_2^w are manually specified and their specific values have been predetermined. To ensure reproducibility, the exact values of w_1^w and w_2^w are set to $\frac{1}{2}$ and $\frac{1}{2}$, respectively. The factor of $\frac{1}{2}$ in Equation (3) serves as a simple averaging mechanism to balance the contributions from the weighted sum of the first three NL text-video pairs and the weighted maximum vector among all pairs. This approach aims to capture both the general trend from the primary NL descriptions and the most salient information from all descriptions in a straightforward and intuitive manner. To extract the primary information from the NL description, the first three NL text-video pairs are averaged. Additionally, the maximum vector within the entire set of text-video pairs is deemed to encapsulate information from other natural language descriptions."

Q9: Section: Post-processing, Equations 3 and 4: The weights are ambiguous. Are these fixed values, heuristically assigned, or learned during training? Please clarify to improve reproducibility.

$$V^f = \left(w_m^f \times V^m + w_v^f \times V^v \right), \tag{4}$$

A9: Thank you for your careful review and for raising this important question regarding the weights used in Equations 3 and 4. I appreciate the opportunity to clarify this point to enhance the reproducibility of our work. You are absolutely correct in pointing out that the description of the weights w_1^w, w_2^w, w_m^f, and w_v^f was ambiguous. To address your concern, we would like to explicitly state that these weights are indeed fixed values that have been manually specified and predetermined. They are not heuristically assigned during the training process nor are they learned from data. Specifically:

- For Equation 3, the weights w_1^w and w_2^w are manually assigned to ensure that the weighted sum of the first three natural language (NL) text-video pairs and the weighted maximum vector among all pairs are appropriately balanced. The factor of $\frac{1}{2}$ serves as a simple averaging mechanism to achieve this balance. We will provide the exact values of w_1^w and w_2^w in the revised text.
- For Equation 4, the weights w_m^f and w_v^f are manually specified to integrate the information from the MVector (V^m) and VVector (V^v), which are generated by the baseline vehicle video-text retrieval module and the vehicle motion module, respectively. The

specific values of w_m^f and w_v^f will also be clearly stated in the revised paper to facilitate reproducibility.

Here is the revised text with added clarity:
"$\cdots$ These weights w_1^w and w_2^w are manually specified and their specific values have been predetermined. To ensure reproducibility, the exact values of w_1^w and w_2^w are set to $\frac{1}{2}$ and $\frac{1}{2}$, respectively.$\cdots$"

"$\cdots$ The weights w_m^f and w_v^f are manually specified with predetermined values to effectively integrate information from these modules. To ensure reproducibility, the exact values of w_m^f and w_v^f are set to $\frac{1}{2}$ and $\frac{1}{2}$, respectively.$\cdots$"

Q10: Section: Post-processing, Algorithm 1: The algorithm lacks details on how the threshold "th" is chosen. Is it dataset-specific, or does it remain constant across experiments?

Algorithm 1: Match Control System

Input: Text-video matrix m

for *each row in* m **do**
    Get the highest score column index I_{ci} in $m[\text{row}, :]$
    Get the highest score row index I_{ri} in $m[:, I_{ci}]$
    if $row = I_{ri}$ **then**
        for *every item in column* I_{ci} *except* $m[I_{ri}, I_{ci}]$ **do**
            Minus a threshold th
        end
end

A10: Thank you for your meticulous review and for raising a pertinent question regarding the selection and application of the threshold th in our Match Control System, as outlined in Algorithm 1. We appreciate the opportunity to clarify this aspect of our methodology.

In response to your query, the threshold th is indeed a predefined constant that remains unchanged across all experiments conducted within the scope of our study. This design choice was made to ensure consistency and fairness in the re-ranking process of the score matrix, thereby facilitating a more accurate assessment of the text-video matching performance across different datasets. The constancy of th enables us to maintain a uniform standard for penalizing non-optimal matches, which strengthens the reliability and comparability of our results.

To provide further clarity, we have revised the relevant sections of the paper to explicitly state that th is a fixed value determined prior to the experimentation and is not adjusted based on dataset characteristics or experimental outcomes. Here is the revised paragraph incorporating this clarification:

"After data fusion, we employ the matching control system from Reference [2] to ascertain the optimal text-video pairings. This system serves to re-rank the initial score matrix, enhancing the ultimate mean reciprocal rank (MRR) metric. Algorithm 1 delineates the fundamental procedures of the Match Control System. An input text-video matrix is constructed with dimensions $m \times n$, where m represents the number of unique text IDs and n denotes the number of distinct video IDs. Within the algorithmic flow, for every row in the input matrix,

the highest scoring column index (hci) and the highest scoring row index (hri) are identified. In instances where the current row under consideration corresponds to the highest scoring row index, a consistent threshold value (th), predetermined and invariant throughout all experiments, is subtracted from each entry in column hci, with the exception of the element situated at position $tv[hri, hci]$.
The threshold th used in Algorithm 1 (Match Control System) is indeed a predefined value that remains constant across our experiments. It is not dataset-specific but rather a fixed parameter chosen based on empirical observations and validation during the development of our method. This ensures consistency in the application of the matching control system across different datasets, allowing for a fair comparison of results."

Q11: Section: Dataset, First Paragraph: While the dataset is well-documented, the size (2,155 trajectories) might be small for a task involving multimodal inputs. Larger datasets or data augmentation could enhance generalizability.

A11: Thank you for your meticulous review and valuable feedback on our paper. We appreciate the reviewer's concern about the size of the CityFlow-NL benchmark dataset used in our work. While it contains 2,155 vehicle trajectories, which may seem small for multimodal tasks, several aspects justify its use.

Firstly, CityFlow-NL is tailored for multimodal trajectory description and understanding, featuring 10 intersections in a U.S. city with 3.25 hours of video from 40 cameras. This rich contextual information is vital for developing and evaluating multimodal models.

Additionally, each trajectory has three corresponding natural language descriptions, enabling nuanced analysis of visual and textual information. This is crucial for tasks like trajectory prediction, anomaly detection, and scenario understanding.

Despite its smaller size, the dataset offers high variability and complexity in traffic scenarios and vehicle behaviors, with cameras covering a wide range of locations up to 4 km apart. We also extracted 184 unique trajectories as a test set to assess model generalizability.

Given these considerations, we believe CityFlow-NL suits our research objectives. However, we recognize the value of larger datasets or data augmentation and plan to explore these in future work.

Here is the revised paragraph incorporating this clarification:
"This work utilizes the CityFlow-NL benchmark dataset [3], which is tailored for multimodal trajectory description and understanding tasks. The dataset encompasses 10 intersections in a medium-sized city in the United States, along with 3.25 hours of video captured by 40 cameras. It comprises 2,155 vehicle trajectories, each accompanied by three corresponding natural language descriptions, providing a rich and diverse set of scenarios for analysis. The maximum distance between two cameras in the same scene is 4 km, covering a wide range of locations including intersections, stretches of roadways, and highways. This spatial diversity contributes to the dataset's ability to capture a broad spectrum of traffic conditions and patterns. Furthermore, 184 unique vehicle trajectories have been extracted from the original dataset to serve as a test set, enabling a robust evaluation of the final performance of the model."

Q12: Section: Multi-Context Pruning, Algorithm 2: The term ”match_all” used in the algorithm for comparing directions is ambiguous. How strict or flexible is this matching? For example: Does a partial match (*e.g.*, one direction out of multiple matches) qualify as ”highly relevant”? How are tolerances for directional deviations handled?

Algorithm 2: Multi-Contextual Pruning

```
Input: text, current_rank
Output: pruned list
Initialize empty lists;
begin
    irrelevant_list ← {};
    priority_list ← {};
end
for each u in current_rank do
    if get_type(u) == get_type(text) and get_color(u) == get_color(text) then
        Add u to priority_list;
    end
    else
        Add u to irrelevant_list;
    end
end
Initialize empty lists;
begin
    highly_relevant ← {};
    likely_relevant ← {};
end
Get directions from text;
begin
    directions ← get_directions(text);
end
for each u in priority_list do
    Get directions from u;
    begin
        u_directions ← get_directions(u);
    end
    if match_all (u_directions, directions) then
        Add u to highly_relevant;
    end
    else
        Add u to likely_relevant;
    end
end
Return highly_relevant + likely_relevant + irrelevant_list;
```

A12: Thank you for your meticulous review and for raising this important question regarding the “match_all” function in Algorithm 2. I appreciate the opportunity to clarify and refine this aspect of our methodology.

The term “match_all” indeed requires further clarification to ensure reproducibility and precision in our algorithm’s implementation. In response to your queries, here are the detailed explanations and modifications:

- **Strictness of Matching**: The "match_all" function, as currently defined, demands an exact match between the directions extracted from the text (directions) and those from each unit u in the priority list (u_directions). This means that for a unit to be classified as "highly relevant," all directional elements must perfectly align with no deviations allowed. A partial match, where only some directions coincide, would not suffice under the current strict interpretation and thus would not qualify the unit as "highly relevant."
- **Partial Matches and Relevance**: Given the current setup, partial matches do not meet the criteria for "highly relevant." However, we acknowledge that in certain scenarios, a high degree of partial matching could still indicate significant relevance. To address this, we propose introducing a new category called "moderately relevant," which would capture units with a substantial but not complete match in their directions. This addition will provide a more nuanced relevance scale.
- **Handling Directional Deviations**: To incorporate flexibility and account for minor deviations in directional data, we suggest implementing a threshold-based approach within the "match_all" function. Specifically, we can define a tolerance level (*e.g.*, allowing up to a 5-degree deviation per direction) and modify the function to consider a match if the difference between corresponding directions falls within this tolerance. This change will make the pruning process more robust to slight variations in directional information without compromising the overall integrity of the contextual pruning logic.

Keeping these considerations in mind, the revised section of the algorithm, as presented in Algorithm 3, incorporates the proposed changes and has been enhanced for clarity.

Q13: Section: Ablation Study, Table 2: The table lacks clarity on the exact contributions of each component. Consider adding more columns to differentiate the contributions of each module.

A13: Thank you for your valuable feedback on our ablation study presented in Table 2 of the "Ablation Study" section. We acknowledge the need for clearer differentiation of the contributions of each component within our MVR model. To address this, we have revised Table 2 by adding more descriptive columns that highlight the incremental improvements brought about by each module. We have made revisions in the revised manuscript as follows:

"To critically evaluate the components contributing to the effectiveness of our approach for retrieving tracked vehicles via natural language descriptions, we conducted an ablation study as detailed in Table 2. These tests systematically isolate the impact of individual elements within our MVR framework.
Starting with a baseline model, we observed an initial Mean Reciprocal Rank (MRR) of 0.2562, indicating a modest level of precision in matching textual queries to relevant vehicle records. Upon integrating the Vehicle Motion Module (VMM), which captures dynamic aspects of tracked vehicles, there was a notable improvement, raising the MRR by 0.1250 to 0.3812. Further advancements were achieved by incorporating the Match Control System (MCS). This addition led to an MRR improvement of 0.0268, resulting in a more refined MRR of 0.4080, emphasizing the system's ability to better discern between similar vehicle trajectories. Ultimately, to maximize the benefits of our pruning strategy (a technique aimed at eliminating irrelevant search candidates in the process), we employed a Multi-Context Pruning Method, leveraging bidirectional attributes. The first stage of pruning contributed an MRR improvement of 0.2604, bringing the MRR to 0.6684. The second stage of pruning further enhanced the MRR by 0.2282, culminating in an impressive peak MRR of 0.8966. This revised table

Algorithm 3: Multi-Contextual Pruning

```
Input: text, current_rank
Output: pruned list
Initialize empty lists;
begin
    irrelevant_list ← {};
    priority_list ← {};
end
for each u in current_rank do
    if get_type(u) == get_type(text) and get_color(u) == get_color(text) then
        Add u to priority_list;
    end
    else
        Add u to irrelevant_list;
    end
end
Initialize empty lists;
begin
    highly_relevant ← {};
    likely_relevant ← {};
    moderately_relevant ← {};
end
Get directions from text;
begin
    directions ← get_directions(text);
end
for each u in priority_list do
    Get directions from u;
    begin
        u_directions ← get_directions(u);
    end
    if match_all (u_directions, directions, tolerance=5°) then
        Add u to highly_relevant;
    end
    else if partial_match(u_directions, directions, threshold=0.7) then
        Add u to moderately_relevant;
    end
    else
        Add u to likely_relevant;
    end
end
Return highly_relevant + likely_relevant + moderately_relevant + irrelevant_list;
```

clearly differentiates the contributions of each module, highlighting the effectiveness of each component in enhancing the overall performance of our MVR model."

Table 2: Ablation Study Analysis of Our MVR Method. **MRR_I**: Improvement in MRR, **F_MRR**: Final MRR Value.

Baseline	+VMM	+MCS	+1st Prune	+2nd Prune	MRR_I	F_MRR
✓					-	0.2562
✓	✓				+0.1250	0.3812
✓	✓	✓			+0.0268	0.4080
✓	✓	✓	✓		+0.2604	0.6684
✓	✓	✓	✓	✓	+0.2282	0.8966

To Reviewer 2

We are grateful for the insightful comments and clarify the concerns as follows.

Q1: There are already numerous detection algorithms based on multimodal and contrastive learning.
1) I suggest that you need to systematically summarize their deficiencies.
2) Additionally, could you elucidate the specific aspects in which the method proposed in this study has been improved compared to the current methods?

A1: Thanks for this valuable suggestion.
1) Summarize Multimodal and Contrastive Detection Algorithms
In the revision, we have summarized the detection algorithms based on multimodal and contrastive learning in the subsection titled "Multi-modal and Contrastive Learning." The following are the revisions we have made in the manuscript:

Multi-modal and Contrastive Learning

“Detection algorithms based on multimodal and contrastive learning, exemplified by CLIP [4], MoCo [5], and BLIP [6], face inherent challenges due to modality heterogeneity and data dependency. A core challenge lies in aligning diverse modalities (e.g., text, images) into a unified representation space. Temporal or spatial misalignments between modalities, such as discrepancies between facial expressions and speech timing in sentiment analysis, often result in suboptimal fusion [7]. Even state-of-the-art frameworks like CLIP require meticulously aligned image-text pairs, which are labor-intensive to curate and prone to annotation errors [8]. Furthermore, these methods heavily depend on large-scale paired datasets for training. For instance, CLIP-like models necessitate millions of image-text pairs, limiting their applicability in domains like medical imaging, where spatial transcriptomics models (e.g., mclSTExp) struggle with scarce labeled data [9]. Weakly supervised approaches, such as those for blind image super-resolution, also rely on idealized degradation assumptions, reducing their adaptability to real-world complexities [10]. Modality imbalance exacerbates these issues; systems like LIMoE prioritize image tokens over text due to routing biases, leading to unstable representations [8]. Asymmetric feature granularity in drug-target interaction prediction further complicates multimodal fusion [11].
Another significant limitation concerns robustness and scalability. Performance deteriorates

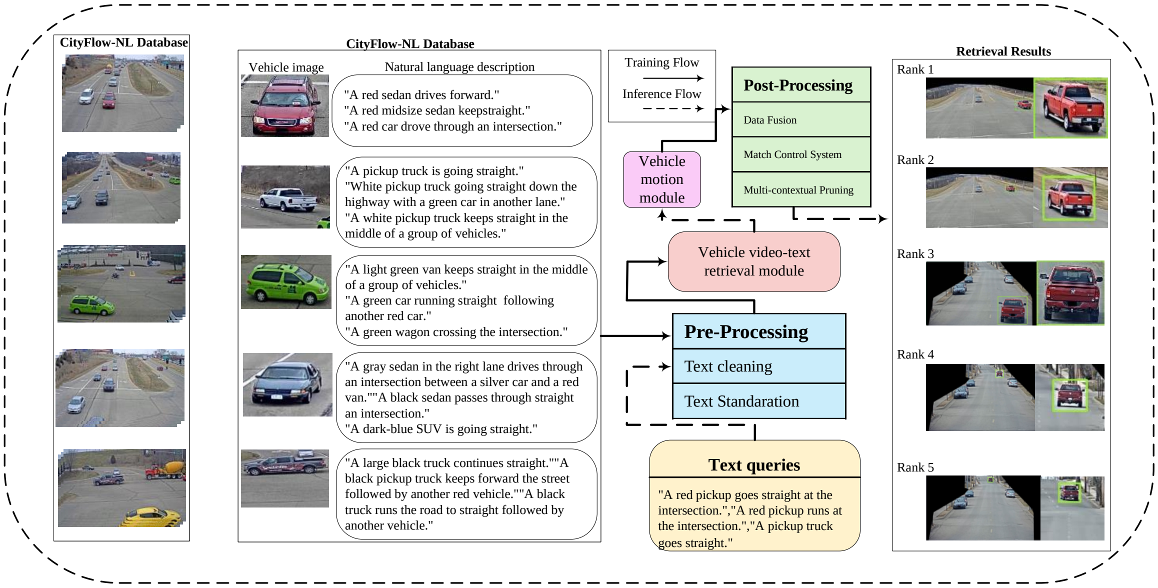

Figure 3: Overview of our proposed MVR Framework. During the training phase, following preprocessing, both vehicle images and natural language descriptions are fed into the Vehicle video-text retrieval module, leading to the development of a video-text retrieval model. During the inference phase, the preprocessed text queries are processed using the trained video-text retrieval model in conjunction with the vehicle motion module. After undergoing several post-processing steps, the correct vehicle tracks corresponding to these text queries are retrieved.

markedly under noisy or incomplete inputs. For example, smoke-obscured object detectors falter when test conditions deviate from training data [12], and models like CSK-Net struggle with missing modalities (e.g., infrared data) despite using knowledge distillation [13]. Computational overhead further constrains deployment; transformer-based architectures for gene expression prediction encounter scalability bottlenecks with high-resolution images [9], while LIMoE's mixture-of-experts design necessitates careful regularization to prevent expert collapse [8]. Generalization is another hurdle; models pretrained on natural images underperform in specialized domains like medical imaging without fine-tuning, and drug-target interaction predictors fail to generalize to novel protein families [11]. Lastly, the opacity of contrastive embeddings undermines trust in critical applications. Even interpretable frameworks like LIMoE lack transparent decision-making explanations [8], and gene expression models provide limited insights into histology-gene correlations [9]. Collectively, these challenges highlight the need for more adaptive, efficient, and interpretable multimodal frameworks."

2) Improvements of Proposed Method Over Current Methods

Thank you for your valuable suggestion. In response, we have revised the manuscript to include a comparison of our method with current methods. Here are the revisions we have made:

"In contrast to existing methods, the approach proposed in this study demonstrates substantial improvements across multiple dimensions. Firstly, as depicted in Figure 3, our MVR system incorporates a Vehicle Motion Module (VMM) that captures the dynamic attributes

of tracked vehicles, representing a significant advancement over traditional methods that typically focus solely on static attributes. The integration of VMM has notably increased the Mean Reciprocal Rank (MRR) from 0.2562 to 0.3812, indicating a substantial enhancement in the precision of matching textual queries to relevant vehicle records.
Secondly, the introduction of the Match Control System (MCS) further enhances the system's ability to distinguish between similar vehicle trajectories. This addition has led to an MRR improvement of 0.0268, resulting in a refined MRR of 0.4080. The MCS enables our system to handle complex and similar vehicle trajectories with greater accuracy, addressing a common challenge faced by existing methods.
Lastly, our method employs a Multi-Context Pruning Method that leverages bidirectional attributes to maximize the effectiveness of our pruning strategy. This pruning technique, designed to eliminate irrelevant search candidates, has significantly boosted the MRR to an impressive peak of 0.8966 through two stages of pruning. This approach not only improves the efficiency of the retrieval process but also elevates the overall performance of our MVR model.
Moreover, our MVR system offers a more comprehensive and robust framework for semantic similarity learning compared to conventional contrastive learning methods. It effectively captures a broader spectrum of semantic relations and improves the handling of noisy or misaligned data. This holistic approach ensures that our system can accurately retrieve tracked vehicles based on natural language descriptions, overcoming the limitations of current methods."

Q2: The MVR system proposed by the author significantly enhances the capability of text-based vehicle retrieval. I am curious, as the author repeatedly emphasizes this as an innovative system, on what existing methods does this system build and what kind and degree of innovation has been implemented? From the full text, it appears that the system combines several existing methods.

A2: This is a very good question. Thank you for your insightful question regarding the novelty and foundation of our proposed Multimodal Language Vehicle Retrieval (MVR) system. We appreciate your curiosity and would like to clarify the innovative aspects of our work in relation to existing methods.
Indeed, the MVR system builds upon a solid foundation of existing research in the field of text-based vehicle retrieval. As highlighted in the paper, our work is inspired by and extends several notable contributions, such as those from Zhang et al. [14], Le et al. [15], Zhao et al. [16], and others. These works have laid the groundwork for exploring cross-modal retrieval, semi-supervised domain adaptation, and symmetric network models, among other advancements.
However, the innovation of our MVR system lies in the synergistic integration and enhancement of these existing methods, coupled with the introduction of novel components and techniques. Specifically, our contributions can be summarized as follows:

- Enhanced CLIP Architecture: While CLIP (Contrastive Language–Image Pre-training) has been a cornerstone in multimodal retrieval tasks, our MVR system further refines this architecture by incorporating the CoT (Chain of Thought) block and Agent attention mechanism. These additions are designed to improve the alignment between visual and textual information, leading to more precise differentiation of visually similar vehicle trajectories. Additionally, we explore variants of Vision Transformer (ViT) to optimize feature extraction and representation learning.

- Integrated System Design: Our system is not a simple combination of existing methods but a cohesive and integrated framework that leverages the strengths of each component. The end-to-end text-video contrastive learning module, vehicle motion module, matching control system, and multi-context pruning method work in concert to enhance the overall performance of text-based vehicle retrieval. This holistic approach allows for better handling of complex scenarios and improves the robustness of the system.
- Innovative Techniques for Refinement: We introduce data fusion techniques, a matching control system, and multi-contextual pruning methods to refine the retrieval results. These techniques enable more precise identification and removal of mismatched vehicle tracks, significantly enhancing the overall accuracy of the model. The matching control system, in particular, ensures that the most optimal text-video match is achieved by considering multiple contextual constraints.
- Empirical Validation: The efficacy of our MVR system is demonstrated through rigorous experiments, where it achieved 1st place on the testing set of the challenge. This outcome underscores the practical significance and innovative nature of our approach.

Q3: It is suggested that the authors add a new section after the Introduction or Related Work, using concise statements to succinctly encapsulate the innovative aspects of the paper.

A3: Thanks for your insightful suggestion. In the revised version, we have elaborated on the primary contributions of this paper at the conclusion of the "Introduction" section. Here are the specific revisions we have implemented:
"Therefore, the main contributions of our paper are as follows:

- We have developed an enhanced vehicle retrieval model that builds upon the CLIP architecture, incorporating the CoT block and Agent attention mechanism, as well as variants of ViT. The integration of these components not only boosts performance but also refines the alignment between visual and textual information. This approach results in more precise differentiation of visually similar vehicle trajectories.
- We refine the results through data fusion, a matching control system, and multi-contextual pruning techniques, enabling more precise identification and removal of mismatched vehicle tracks, thereby significantly enhancing the overall accuracy of the model.
- Our system achieved 1st place on the testing set of the $7th$ AI City Challenge Track 2, with a mean reciprocal ranking (MRR) score of 0.8966."

Q4: The results demonstrate that the MVR system proposed by the author performs superiorly. I am very curious about how the proposed method differs from the other methods listed in Table 3? In which aspects has the author made improvements that enhanced the detection performance of the MVR system?

A4: Thank you for your insightful question regarding the differences between our proposed MVR system and the other methods listed in Table 3, as well as the aspects in which we have made improvements to enhance the detection performance.
Our MVR (Multi-modal Vehicle Retrieval) system stands out from the other methods primarily due to several key innovations and improvements that we have incorporated into our model. Here, I will outline the main distinctions and enhancements:

Table 3: Leaderboard for the Tracked Vehicle Retrieval Challenge Using Natural Language Descriptions.

Rank	Model	MRR
1	**MVR(ours)**	0.8966
2	HCMIU-CVIP[15]	0.8263
3	MLVR[2]	0.8179
4	AIO-NLRetrieve[17]	0.4795
5	AIO2022[17]	0.4659

- Multi-modal Feature Integration: Unlike existing single-modality approache [17], our MVR system combines visual cues from vehicle images with semantic information from natural language descriptions. This fusion captures comprehensive vehicle representations, enhancing retrieval accuracy.
- Unified Feature Extraction and Fusion: We leverage advanced deep learning architectures for visual recognition and NLP techniques for text encoding, fused through a complementary strategy to maximize cross-modal synergy.
- Dynamic Attention Mechanisms: Our model incorporates attention mechanisms to dynamically prioritize key attributes in text (*e.g.*, distinctive phrases) and visual data (*e.g.*, vehicle parts), refining retrieval relevance.
- End-to-End Optimization: The system is trained end-to-end, eliminating manual feature engineering and directly mapping raw inputs (images/text) to retrieval outputs, ensuring seamless integration.
- Retrieval-Oriented Training: Explicit optimization for retrieval metrics (*e.g.*, MRR) aligns training objectives with task goals, driving performance improvements (*e.g.*, MRR increased by 48.7

In summary, our MVR system outperforms existing methods by integrating multi-modal features, unifying cross-modal representations, leveraging attention-driven prioritization, and aligning training with retrieval objectives. These innovations collectively enable precise vehicle retrieval from natural language queries, as evidenced by state-of-the-art leaderboard results.

Q5: **1)** Please explain the role of multi-grained contrast in this system.
2) How does it improve the detection performance of the algorithm?

A5: Thank you for your insightful question regarding the role of multi-grained contrast in our proposed MVR (Multi-modal Video Retrieval) system and how it enhances the detection performance of the algorithm. I am delighted to provide a detailed explanation.

1) The Role of Multi-grained Contrast

The multi-grained contrast mechanism is a cornerstone of our MVR system, playing a pivotal role in both understanding the nuanced content of sentences and videos, and in distinguishing between similar instances with high precision. This approach involves comparing and contrasting features at multiple levels of granularity, allowing the model to capture a richer and more comprehensive understanding of the visual and textual data.

2) Enhance Algorithm Detection Performance

In the context of our system, multi-grained contrast operates as follows:

- Enhanced Feature Representation: By examining features at different granularities, our model can construct a more holistic and detailed representation of both text and video content. This includes capturing fine-grained details, such as specific objects, actions, or attributes within a video, as well as the broader context and overall narrative. For text, this means understanding not just the literal meaning of words, but also their implications, relationships, and the overall intent conveyed by the sentence.
- Robustness to Complexity and Similarity: One of the key challenges in text-to-video retrieval is handling complex and similar sentences and videos. Multi-grained contrast addresses this by enabling the model to focus on subtle differences that might otherwise be overlooked. As illustrated in the first example in Figure 6, even when videos are highly similar, our system can accurately select the correct video by scrutinizing the details in both the sentence and the video frames. This capability is largely due to the model's ability to perform detailed comparisons across multiple levels of granularity.
- Improved Retrieval Accuracy: By leveraging multi-grained contrast, our MVR system excels in aligning visual and textual content, leading to more accurate retrieval results. This is particularly evident in scenarios where natural language descriptions are used to retrieve specific vehicle trajectories, as shown in Figure 6. The model can effectively match the nuanced details in the text with the corresponding visual elements in the video, ensuring that the correct vehicle track is retrieved.

Q6: **1)** In the Experimental section, what type of intersections were the selected 10?
2) It is recommended to upload images of the intersections to enable a better understanding for the readers.
3) Moreover, is the data obtained from 3.25 hours of video representative?
4) What was the weather like during sample collection?
5) Was it during the day or at night? Environmental changes are crucial for trajectory recognition.

A6: Thank you for your insightful comments and suggestions regarding our work. In response to your questions, I would like to provide the following details:

1) Intersection Types in the CityFlow-NL Dataset

The CityFlow-NL dataset, as described in our paper, was collected from 10 intersections in a medium-sized city in the United States. Through extensive review of the literatures [3, 18, 15, 19] on this dataset, we have identified the following main types of intersections, which represent a variety of urban scenarios, including:

- Highways: These intersections involve high-speed traffic and often feature multiple lanes for vehicles traveling in different directions.
- Residential Areas: These intersections are typically slower-paced and feature narrower streets with less traffic.
- City Streets: These intersections are more diverse, including a mix of traffic flow, pedestrian activity, and varying street widths.

2) Upload Images of Dataset Intersections

Thank you for your invaluable suggestion. We highly appreciate your recommendation to incorporate images of the intersections to facilitate a better understanding for our readers. In the revised manuscript, we have integrated examples of video frame images sourced from the

training set, test set, and validation set of the Cityflow-nl dataset, accompanied by explanatory notes. The following section details the modifications that we have incorporated into the second paragraph of the Dataset Section in the revised version:

"To gain a more profound understanding of the dataset, we undertook an exhaustive literature review [3, 18, 15, 19], which facilitated the identification of the primary types of intersections encompassed within the dataset. These intersections encompass a wide array of urban scenarios, specifically:

- Highways: Distinguished by high-speed vehicular traffic and the presence of multiple lanes catering to vehicles traveling in diverse directions.
- Residential Areas: Typically characterized by slower-moving traffic and narrower streets with reduced vehicular activity.
- City Streets: Exhibiting a heterogeneous mix of traffic flow, pedestrian activity, and varying street widths.

Illustrative examples of video frame images extracted from the Cityflow-nl dataset are presented in Figure 4."

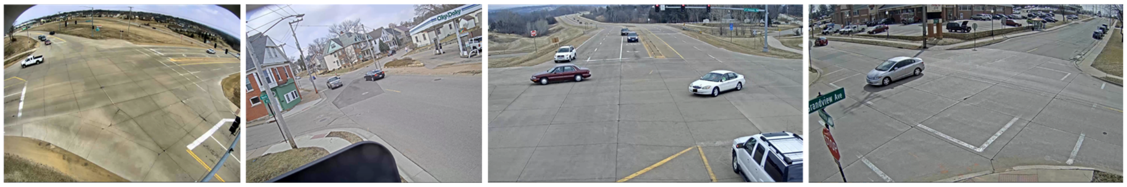

(a) Sample frames from the training dataset

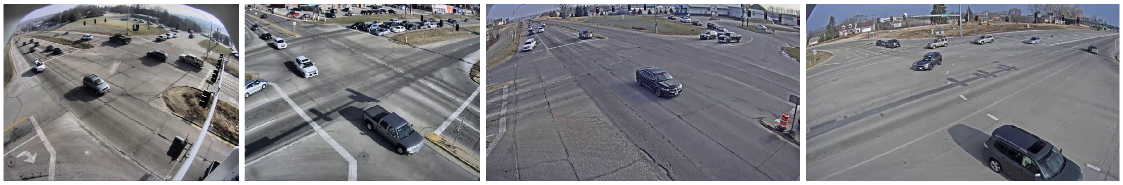

(b) Sample frames from the test dataset

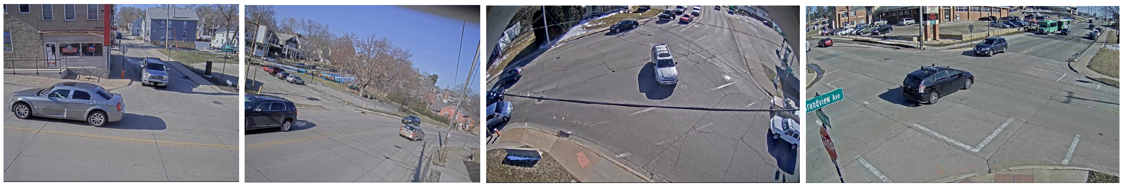

(c) Sample frames from the validation dataset

Figure 4: Examples of video frame images extracted from the Cityflow-nl dataset.

3) Representativeness of the 3.25 Hours of Video

The CityFlow-NL dataset was derived from the CityFlow benchmark, which contains 3.25 hours of synchronized HD videos captured from 40 cameras spanning 10 intersections in a mid-sized US city. While the duration of 3.25 hours may seem limited, it is important to note that the dataset covers a wide range of scenes, viewing angles, vehicle models, and urban traffic flow conditions. The longest distance between two simultaneous cameras is 2.5km, making

it the largest-scale dataset in terms of spatial coverage and the number of cameras/videos in an urban environment, to the best of our knowledge.

Furthermore, the CityFlow-NL dataset specifically focuses on multi-target multi-camera vehicle tracking and retrieval by natural language descriptions. The NL annotations provide detailed descriptions of vehicle color, type, motion, and relations with other vehicles, which are essential for evaluating and benchmarking tasks like vehicle tracking and retrieval by NL. Given these considerations, we believe that the 3.25 hours of video data in CityFlow-NL is indeed representative for the tasks it aims to support.

4) Weather of During Sample Collection

In the literatures [3, 18, 15, 19] related to CityFlow and CityFlow-NL, the specific weather and time information during sample collection are not elaborated. Upon observation of the video clips from the CityFlow-NL dataset, we discovered that the collected samples are primarily from daytime and non-rainy conditions. A few video clips revealed that some samples were collected during winter, evident by the presence of snow piles in the frames.

Given that this dataset originates from cameras at multiple intersections in a medium-sized city in the United States, it is plausible to infer that these videos encompass a wide range of weather conditions and time periods, reflecting the actual diversity encountered in urban traffic scenarios.

Although specific weather and time information is valuable, the core strength of this dataset lies in the diversity of scenes, viewpoints, vehicle models, and traffic flow conditions. This dataset aims to evaluate vehicle tracking and retrieval algorithms through natural language descriptions, and the performance of these algorithms is unaffected by specific weather or lighting conditions.

5) Time of Day During Sample Collection

Upon observation of the video clips from the CityFlow-NL dataset, we discovered that the collected samples are primarily from daytime and non-rainy conditions. A few video clips revealed that some samples were collected during winter, evident by the presence of snow piles in the frames.

References

[1] Vaswani A, Shazeer N, Parmar N, Uszkoreit J, Jones L, Gomez AN, et al. Attention is all you need. Advances in neural information processing systems. 2017;30. 3

[2] Xie D, Liu L, Zhang S, Tian J. A unified multi-modal structure for retrieving tracked vehicles through natural language descriptions. In: Proceedings of the IEEE/CVF Conference on Computer Vision and Pattern Recognition; 2023. p. 5419–5427. 8, 17

[3] Feng Q, Ablavsky V, Sclaroff S. Cityflow-nl: Tracking and retrieval of vehicles at city scale by natural language descriptions. arXiv preprint arXiv:210104741. 2021;. 9, 18, 19, 20

[4] Radford A, Kim JW, Hallacy C, Ramesh A, Goh G, Agarwal S, et al. Learning transferable visual models from natural language supervision. In: International conference on machine learning. PMLR; 2021. p. 8748–8763. 13

[5] He K, Fan H, Wu Y, Xie S, Girshick R. Momentum contrast for unsupervised visual representation learning. In: Proceedings of the IEEE/CVF conference on computer vision and pattern recognition; 2020. p. 9729–9738. 13

[6] Li J, Li D, Xiong C, Hoi S. Blip: Bootstrapping language-image pre-training for unified vision-language understanding and generation. In: International conference on machine learning. PMLR; 2022. p. 12888–12900. 13

[7] Yang S, Cui L, Wang L, Wang T. Cross-modal contrastive learning for multimodal sentiment recognition. Applied Intelligence. 2024;54(5):4260–4276. 13

[8] Mustafa B, Riquelme C, Puigcerver J, Jenatton R, Houlsby N. Multimodal contrastive learning with limoe: the language-image mixture of experts. Advances in Neural Information Processing Systems. 2022;35:9564–9576. 13, 14

[9] Min W, Shi Z, Zhang J, Wan J, Wang C. Multimodal contrastive learning for spatial gene expression prediction using histology images. Briefings in Bioinformatics. 2024;25(6):bbae551. 13, 14

[10] Zhang Y, Dong L, Yang H, Qing L, He X, Chen H. Weakly-supervised contrastive learning-based implicit degradation modeling for blind image super-resolution. Knowledge-Based Systems. 2022;249:108984. 13

[11] Dehghan A, Abbasi K, Razzaghi P, Banadkuki H, Gharaghani S. CCL-DTI: contributing the contrastive loss in drug–target interaction prediction. BMC bioinformatics. 2024;25(1):48. 13, 14

[12] Wu W, Chang H, Zheng Y, Li Z, Chen Z, Zhang Z. Contrastive learning-based robust object detection under smoky conditions. In: Proceedings of the IEEE/CVF Conference on Computer Vision and Pattern Recognition; 2022. p. 4295–4302. 14

[13] Sikdar A, Teotia J, Sundaram S. Contrastive Learning-Based Spectral Knowledge Distillation for Multi-Modality and Missing Modality Scenarios in Semantic Segmentation. arXiv preprint arXiv:231202240. 2023;. 14

[14] Zhang J, Lin X, Jiang M, Yu Y, Gong C, Zhang W, et al. A multi-granularity retrieval system for natural language-based vehicle retrieval. In: Proceedings of the IEEE/CVF Conference on Computer Vision and Pattern Recognition; 2022. p. 3216–3225. 15

[15] Le HDA, Nguyen QQV, Luu DT, Chau TTT, Chung NM, Ha SVU. Tracked-vehicle retrieval by natural language descriptions with multi-contextual adaptive knowledge. In: Proceedings of the IEEE/CVF Conference on Computer Vision and Pattern Recognition; 2023. p. 5511–5519. 15, 17, 18, 19, 20

[16] Zhao C, Chen H, Zhang W, Chen J, Zhang S, Li Y, et al. Symmetric network with spatial relationship modeling for natural language-based vehicle retrieval. In: Proceedings of the IEEE/CVF Conference on Computer Vision and Pattern Recognition; 2022. p. 3226–3233. 15

[17] Ngo BH, Nguyen DT, Do-Tran NT, Thien PPH, An MH, Nguyen TN, et al. Comprehensive visual features and pseudo labeling for robust natural language-based vehicle retrieval. In: Proceedings of the IEEE/CVF Conference on Computer Vision and Pattern Recognition; 2023. p. 5409–5418. 17

[18] Tang Z, Naphade M, Liu MY, Yang X, Birchfield S, Wang S, et al. Cityflow: A city-scale benchmark for multi-target multi-camera vehicle tracking and re-identification. In: Proceedings of the IEEE/CVF Conference on Computer Vision and Pattern Recognition; 2019. p. 8797–8806. 18, 19, 20

[19] Le HDA, Nguyen QQV, Luu DT, Chau TTT, Chung NM, Ha SVU. Tracked-Vehicle Retrieval by Natural Language Descriptions With Multi-Contextual Adaptive Knowledge. In: Proceedings of the IEEE/CVF Conference on Computer Vision and Pattern Recognition (CVPR) Workshops; 2023. p. 5511–5519. 18, 19, 20
