# Supplementary material for: A novel multi-modal retrieval framework for tracking vehicles using natural language descriptions
Source: PLoS One. 2025 Aug 11;20(8):e0327468. doi: 10.1371/journal.pone.0327468 (PMC12338829; doi:10.1371/journal.pone.0327468)
Supplement: Attachment — Submitted filename: Response_to_Reviewers_auresp_2.pdf [file pone.0327468.s002.pdf]

Authors' Response of PONE-D-24-43459.R2

Changhao Zhang, Zhandong Liu*, Ke Li, Yong Li, Xiangwei Qi, Nan Ding,

April 14, 2025

To Associate Editor and Reviewers

We sincerely thank you for your valuable feedback and constructive suggestions on the manuscript titled "A Novel Multimodal Retrieval Framework for Tracking Vehicles Based on Natural Language Descriptions." We have carefully considered all the comments and revised the manuscript accordingly. Below are our point-by-point responses to the reviewers' comments, along with the corresponding modifications made to the manuscript. To highlight the main revisions, key changes in the revised version have been prominently marked.

To Reviewer 1

We are grateful for your valuable support.

To Reviewer 2

We appreciate the reviewer's insightful comment regarding the temporal representativeness of the dataset. Below is our detailed response, along with the proposed revisions to the manuscript.

Q1: While the author explains that the 3.25-hour dataset is sufficiently large, sample size is only one aspect of representativeness. To ensure the sample is truly representative and to avoid randomness, data should be collected from multiple different dates, even if only for one hour each. Were the 3.25 hours of data all from the same day? The author should clarify this point and discuss the representativeness of the data from this perspective.

A1: We appreciate the reviewer's insightful comment regarding the temporal representativeness of the CityFlow-NL benchmark dataset [1]. While the 3.25-hour duration is sufficiently large in terms of sample size, we agree that collecting data from multiple dates would further enhance the diversity and reduce potential biases introduced by single-day sampling.

To address this concern, we clarify that the 3.25 hours of video data were not all collected on the same day but spanned multiple days across different seasons, as evidenced by varying weather conditions (e.g., snow presence in winter clips). However, we acknowledge that the exact intervals between the collection dates were not systematically recorded, which is a limitation of this dataset. Despite this, the dataset's spatial diversity (city-scale coverage, varied scenes, and traffic conditions) mitigates some temporal constraints by capturing a wide range of real-world scenarios.

In order to enhance the transparency of our research, we have made revisions to the manuscript and incorporated the following clarification in the Dataset section (Page 15):
"The 3.25-hour video dataset comprises footage aggregated from multiple recording sessions conducted across different days, capturing seasonal variations (e.g., winter scenes with snow piles) and diverse traffic conditions. While the exact collection dates and intervals are not specified due to privacy constraints, the dataset's broad spatial coverage—spanning 10 intersections and 40 cameras—ensures representation of varied environmental and traffic scenarios. "

Q2: The author provides reasonable explanations regarding the factors of weather and nighttime. However, I suggest incorporating relevant discussions into the 'Limitations' and 'Future Research Directions' sections of the conclusion.

A2: We agree with this suggestion and have expanded the Limitations and Future Research Directions sections in the Conclusion (Page 18) to include discussions on weather and nighttime conditions:

Limitations

Despite the strong performance of Multimodal Vehicle Retrieval (MVR), the model's generalization is limited by the current dataset, which primarily consists of daytime and fair-weather samples. Real-world deployment demands robustness against adverse conditions such as low illumination and meteorological interferences like rain and fog. Therefore, validating the system's performance in challenging scenarios, including nighttime and inclement weather, remains essential, as these conditions can hinder visual feature extraction and trajectory tracking.

Future Research Directions

To address these limitations, future work will focus on two main directions: expanding the dataset to include diverse weather conditions and nighttime scenarios, thereby enhancing the system's robustness; and developing synthetic-to-real domain adaptation techniques using physically realistic weather rendering to enable all-weather video retrieval. This will establish MVR as a foundational tool for smart city surveillance systems.

Additional Revisions:

(1) Reference List: We have reviewed the reference list to ensure completeness and correctness. All citations are up-to-date, and no retracted papers are included.

(2) Data Availability: As noted by Reviewer 1, we confirm that all data underlying the findings are fully available in the CityFlow-NL benchmark dataset [1], which is publicly accessible.

References

[1] Feng Q, Ablavsky V, Sclaroff S. Cityflow-nl: Tracking and retrieval of vehicles at city scale by natural language descriptions. arXiv preprint arXiv:210104741. 2021;. 1, 2
